# Palliative Senning in the Treatment of Congenital Heart Disease with Severe Pulmonary Hypertension

**DOI:** 10.5935/abc.20150097

**Published:** 2015-10

**Authors:** Juliano Gomes da Penha, Leina Zorzanelli, Antonio Augusto Barbosa-Lopes, Edimar Atik, Leonardo Augusto Miana, Carla Tanamati, Luiz Fernando Caneo, Nana Miura, Vera Demarchi Aiello, Marcelo Biscegli Jatene

**Affiliations:** Instituto do Coração do Hospital das Clínicas da Faculdade de Medicina da USP, São Paulo, SP – Brazil

**Keywords:** Heart Defects, Congenital, Pulmonary Hypertension, Child, Transposition of the Great Vessels/surgery

## Abstract

**Background:**

Transposition of the great arteries (TGA) is the most common cyanotic cardiopathy, with an incidence ranging between 0.2 and 0.4 per 1000 live births. Many patients not treated in the first few months of life may progress with severe pulmonary vascular disease. Treatment of these patients may include palliative surgery to redirect the flow at the atrial level.

**Objective:**

Report our institutional experience with the palliative Senning procedure in children diagnosed with TGA and double outlet right ventricle with severe pulmonary vascular disease, and to evaluate the early and late clinical progression of the palliative Senning procedure.

**Methods:**

Retrospective study based on the evaluation of medical records in the period of 1991 to 2014. Only patients without an indication for definitive surgical treatment of the cardiopathy due to elevated pulmonary pressure were included.

**Results:**

After one year of follow-up there was a mean increase in arterial oxygen saturation from 62.1% to 92.5% and a mean decrease in hematocrit from 49.4% to 36.3%. Lung histological analysis was feasible in 16 patients. In 8 patients, pulmonary biopsy grades 3 and 4 were evidenced.

**Conclusion:**

The palliative Senning procedure improved arterial oxygen saturation, reduced polycythemia, and provided a better quality of life for patients with TGA with ventricular septal defect, severe pulmonary hypertension, and poor prognosis.

## Introduction

Congenital cardiopathies are the most frequent inborn defects in newborns, representing about 1% of the cases. The transposition of the great arteries (TGA) is the most common cyanotic cardiopathy, with an incidence ranging from 0.2 to 0.4 per 1000 live births^1,2^.

The first proposal for physiologic correction of TGA at the atrial level was described by Albert in 1954. In 1958, Ake Senning performed with success the proposal suggested by Albert, performing the correction at the atrial level using autogenous atrial tissue to construct intracardiac baffles. The use of flaps made of a prosthetic material for intra-atrial correction was first proposed and performed by Mustard in 1964. However, the occurrence of systemic ventricular dysfunction and a high prevalence of arrhythmias as late morbidity factors placed this technique out of use and replaced it with a more physiologic technique, the Jatene procedure^3^.

In 1972, Lindesmith et al^4^ reported for the first time a series of patients with TGA and ventricular septal defect (VSD) with severe pulmonary vascular obstructive disease who underwent a palliative surgery to redirect the flow at the atrial level. The Mustard surgery was the proposed procedure to redirect the pulmonary and systemic venous drainage, maintaining the VSD open. The VSD is maintained open in these patients because its closure is associated with early and late prohibitive mortality, as previously described^4^. From then on, the indications for palliative surgery were widened to include other complex congenital lesions with VSD and pulmonary hypertension (PH)^5^.

The present study aims to report the results of palliative surgical treatment in patients with complex congenital heart disease with PH due to an important intracardiac shunt which was not surgically treated within the period considered safe. It also aims at evaluating the early and late clinical progression with the palliative Senning procedure in this group of patients with contraindication to total surgical correction of the cardiopathy.

## Methods

The study included patients with a diagnosis of TGA with VSD and Taussig-Bing double-outlet right ventricle (DORV), aged up to 11 years, seen by the Pediatric Cardiology and Pediatric Cardiac Surgery teams at *Instituto do Coração*, *Hospital das Clínicas* of the School of Medicine at USP (InCor-HCFMUSP). This was a retrospective study based on the evaluation of medical records between 1991 to 2014. Only patients without indication of definitive surgical treatment of the cardiopathy due to suprasystemic pulmonary pressure were part of the analysis. Patients with a diagnosis of TGA and Taussig-Bing DORV with favorable pulmonary pressure were not included in this study.

The data collected included age and weight at the time of the surgery, preoperative diagnosis, preoperative functional status, palliative procedures prior to the main surgical procedure, type of surgical procedure performed, preoperative hemodynamic status, early and late morbidity including any cardiovascular or pulmonary event and reoperations, late functional status, analysis of lung biopsies, and survival. The statistical analysis was descriptive. (A software was not required since the calculations were performed manually.)

As for the surgical procedure, all patients underwent median sternotomy and opening of the pericardium. The anatomy was verified with careful initial inspection. Following that, an extensive dissection and release of the superior and inferior venae cavae was performed, with the dissection also including the groove between the left and right atria. Pockets were created in the aorta and venae cavae with prolene suture, heparin was infused, and direct cannulation of the aorta and venae cavae was performed. Care was taken to cannulate the venae cavae as distal as possible to facilitate the surgical maneuvers inside the atria. Before full heparinization, a fragment of the lung was removed for histological analysis. This was generally performed with wedge resection of the right upper lobe with the lung inflated. The biopsy was feasible in 16 patients. We used the studies of Heath and Edwards^6^ and Rabinovitch et al^7^ as the criteria for the histological classification of the lung fragments (Table 1).

**Table 1:** Lung biopsy histological classification

**Classification of Rabinovitch et al.**	**Classification of Heath and Edwards**
Grade A: early muscularization of the distal arteries; Grade B: hypertrophy of the arterial wall; Grade C: grade B changes associated with increased proportion of the number of alveoli and arteries.	**Grade 1:** isolated hypertrophy of the media; **Grade 2:** fibrointimal proliferation; **Grade 3:** total occlusion of the lumen by fibrosis; **Grade 4:** plexiform lesions; **Grade 5:** hypertrophy of muscular arteries, cavernous lesions,angiomatoid lesions; **Grade 6: **necrotizing arteritis.

**Figure 1:**
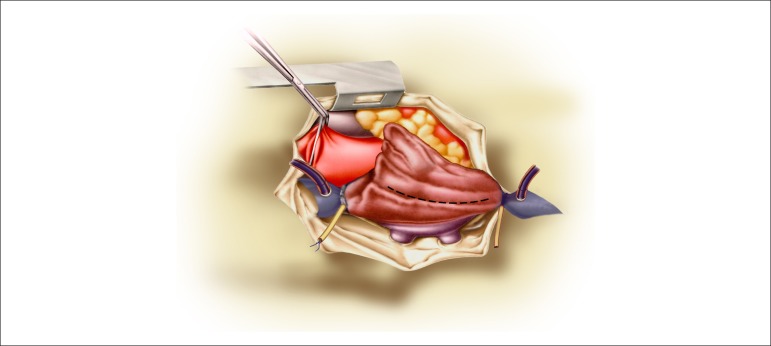
Place of the incision in the right atrium, maintaining a safety margin between the venae cavae and the pulmonary veins.

**Figure 2:**
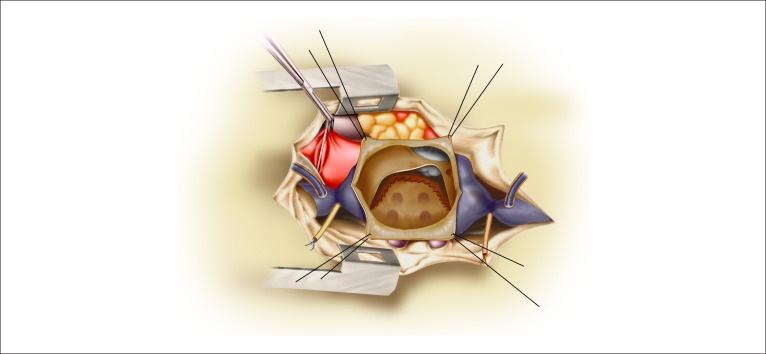
Atrial septal sutures or bovine pericardium and isolation of the pulmonary veins which will be directed to the tricuspid valve and to the aorta.

**Figure 3:**
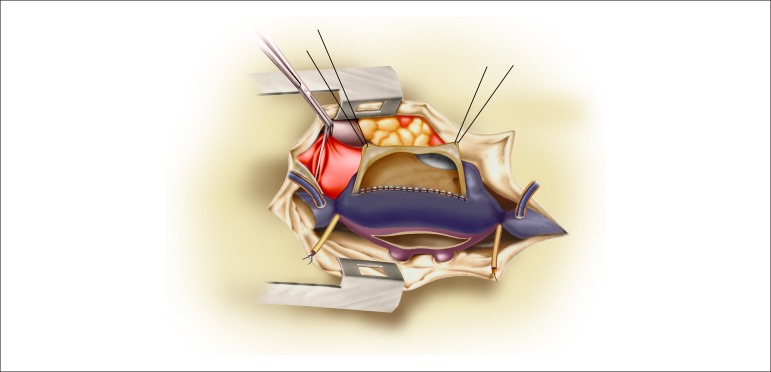
Cava baffle and direction of the venous blood flow to the mitral valve. Opening of the left atrium above the right pulmonary veins.

**Figure 4:**
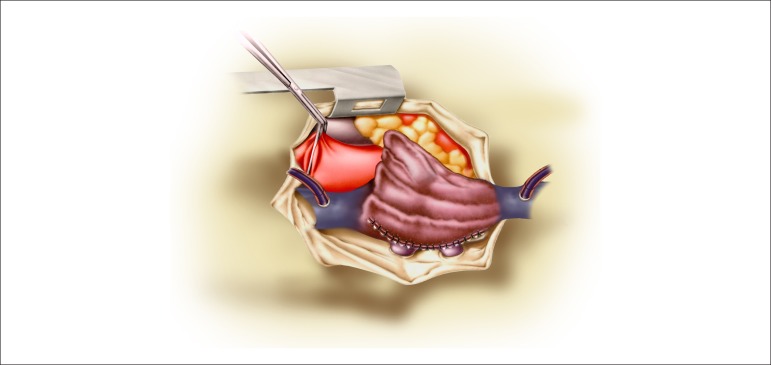
Suture of the edge of the right atrium in place of the opening of the left atrium with redirection of the arterial blood flow to the tricuspid valve.

After full heparinization and cannulation, cardiopulmonary bypass (CPB) was initiated. The ascending aorta was clamped, and the St. Thomas' solution was used for cardioplegia. The cardioplegic solution was initially infused at a rate of 20 mL/kg, and then maintained at 10 mL/kg every 20 to 30 minutes. The target temperature was 28ºC in patients not undergoing total circulatory arrest (TCA) and 20°C in those undergoing TCA. The right atrium was opened with an incision parallel to the interatrial groove, positioned at a distance of about 0.5 to 1 cm from the caval drainage into the right atrium. The atrial septal defect (ASD), the anatomical relations of the tricuspid and mitral valves, and the caval drainage were analyzed. A wide enlargement of the ASD towards the superior and inferior venae cavae was performed and a bovine pericardium patch was sutured covering and isolating the pulmonary veins, leaving the two atrioventricular valves and venae cavae in the same cavity. After that, a cava baffle was constructed by suturing the edge of the lateral wall of the right atriotomy, directing the flow from the venae cavae to the mitral valve. This procedure allows the caval drainage to be directed to the left ventricle which is connected to the pulmonary trunk. An incision was then performed in the left atrium anteriorly to the right pulmonary veins, exposing the left atrium along with the pulmonary veins. After that, the right edge of the left atriotomy was sutured to the left edge of the right atriotomy. With this procedure, the left atrium and pulmonary veins were connected to the tricuspid valve and right ventricle, which is related to the aorta. The VSD was maintained open (Figures 1, 2, 3 and 4).

After redirecting the flow from the atria, the patient was warmed up. Maneuvers were carried out to remove the air from the cavities and for weaning from CPB. The use of modified ultrafiltration became routine after 2011, and intraoperative transesophageal echocardiography was only feasible in children weighing more than 3 kg due to an incompatibility of the probe used in our institution for children weighing less than that. Death in the initial postoperative period was defined as any death occurring within the first 30 days after the surgical procedure or during the same hospitalization.

## Results

From November 1991 to April 2011, a total of 21 patients with a diagnosis of TGA with VSD or Taussig-Bing DORV and severe pulmonary vascular disease were referred to palliative surgical treatment after other types of treatment were precluded. (The last surgery was performed in 2011, but patients were followed up until 2014. This fact results in two different dates in the Results and in the Methods sections). The age of the patients at the time of the surgery ranged from 1 to 130 months (mean 24.6 months and median 16 months), and 30% were aged 12 months or less. Among the 21 patients, 11 were male. The weight of the patients ranged from 2.8 to 30 kg (mean 8.3 kg and median 7.1 kg).

Preoperative functional evaluation according to the New York Heart Association (NYHA) was feasible in 18 patients, and most (83%) were classified as functional class III or IV. The main anatomic diagnoses were TGA with VSD in 17 patients (81%), and Taussig-Bing DORV in 4 patients (19%). Smaller associated defects are shown in Table 2. The Rashkind procedure was performed in 13 of the 21 patients before the surgery, 11 of which had TGA with VSD. One patient in the DORV group who had aortic coarctation had previously undergone isthmoplasty and pulmonary artery banding at the age of 20 days.

**Table 2:** Associated defects

**TGA with VSD**	**17**	**Taussig-Bing DORV**	**4**
Coronary anomaly	6	Coronary anomaly	2
Single left coronary ostium	5	Single left coronary ostium	1
Right coronary artery arising from the circumflex artery	1	Right coronary artery arising from the circumflex artery	1
Situs inversus totalis	1	VSD > 5mm	4
VSD > 5 mm	10	Patent ductus arteriosus	2
Pulmonary valve infundibular stenosis	3	Aortic coarctation	1
Patent ductus arteriosus	5		

TGA: Transposition of the great arteries; VSD: Ventricular septal defect; DORV: Double-outlet right ventricle.

Cardiac catheterization was performed prior to the surgery in all cases. Pulmonary vascular resistance (PVR) with inhaled 100% oxygen ranged from 3.2 to 14 U.m^2^ (mean 8.1 U.m^2^ and median 7.7 U.m^2^). The PVR of 3.2 U.m^2^ was found in a patient with systolic pulmonary artery pressure (SPAP) of 94 mmHg and no response to the oxygen test. Preoperative SPAPs ranged from 41 to 130 mmHg (mean 77.8 mmHg and median 75 mmHg). Oxygen saturation and hematocrit ranged from 40% to 80% (mean 62.1% and median 67%) and 40% to 65% (mean 49.2% and median 50%), respectively.

Length of circulatory assistance ranged between 65 and 170 minutes (mean 113.6 minutes and median 108 minutes). Length of aortic clamping ranged between 50 and 95 minutes (mean 72.5 minutes and median 78.5 minutes). In three patients, TCA with selective cerebral perfusion through the brachiocephalic trunk and deep hypothermia (20°C) were performed, with a mean duration of 52 minutes.

The initial mortality rate was 47% (10 patients). The causes of death were low output in 6 patients, sepsis in 2 patients, and pulmonary hypertensive crisis in the 2 remaining patients. The mean total duration of hospitalization was 15 days (range 1 to 43 days), with a mean duration of hospitalization of 19.1 days in patients discharged from the hospital. Postoperative comorbidities not resulting in death were pulmonary hypertensive crisis (which improved with nitric oxide), pneumonia, acute renal failure (ARF), chylothorax with ligation of the thoracic duct, pulmonary congestion, total atrioventricular block (TAVB), and junctional rhythm (Table 3). Assessments performed 1 year after hospital discharge showed a mean increase in arterial oxygen saturation from 62.1% to 92.5%, and a mean reduction in hematocrit from 49.4% to 36.3%.

**Table 3:** Non-fatal postoperative comorbidities

**PH crisis**	**6**
Pulmonary congestion	4
ARF	2
Chylothorax	2
Pneumonia	1
TAVB	1
Junctional rhythm	1

PH: Pulmonary hypertension; ARF: Acute renal failure; TAVB: Totalatrioventricular block

Lung histological analysis was feasible in 16 patients and was performed with the classifications of Heath and Edwards^6^, and Rabinovitch et al^7^. In 8 patients, grades 3 and 4 pulmonary biopsies were evidenced. Of 10 biopsies in which the classification of Rabinovitch et al^7^ was used, 4 were grade B and 6 were grade C (Table 4). All patients with grades 3 and 4 biopsies were older than 16 months and had a mean pulmonary artery pressure above 45 mmHg.

**Table 4:** Correlation between histology, hemodynamics, and age

**Patient **	**Age (months) **	**SPAP (mmHg) **	**Classification of Heath and Edwards **	**Classification of Rabinovitch et al. **	**PVR (U.m^2^) **
1	16	80	4	C	
2	18	88	1	B	
3	3	94	2	C	
4	73	130	3		
5	23	75	2	C	
6	8	107	2	C	
7	130	89	3		
8	11	41	2	C	5
9	21	53	4		
10	33	67	3	C	9.6
11	1	90	1	B	
12	14	48	2	B	6.6
13	11	59	2	C	
14	23	55	4		6.8
15	8	66	3		8.6
16	38	45	4		

SPAP: Systolic puzlmonary artery pressure; PVR: Pulmonary vascular resistance.

Mean follow-up of the 11 survivors was 6.4 years with a maximum of 19 years. Five patients continue to follow up at our institution while 5 other patients are currently following up in other centers closer to their homes (Manaus, Fortaleza, Brasília, and Salvador). There was 1 case of sudden death at home due to an unknown cause 18 months after hospital discharge.

Functional assessment according to the NYHA was feasible in 10 of the 11 survivors. Functional class improved in all patients (5 class I and 5 class II). Ten had a sinus rhythm, and 1 had a junctional rhythm. None of the patients required definitive pacemaker implantation during follow-up.

Echocardiographic evaluations were performed during follow-up. The ventricular function remained preserved in all patients. One patient presented cava baffle stenosis and 5 presented tricuspid valve insufficiency (3 of moderate degree and 2 of severe degree).

## Discussion

Most patients currently diagnosed with TGA with VSD and Taussig-Bing DORV do not progress to pulmonary vascular disease because total surgical correction is performed early, soon after establishment of the diagnosis, which often occurs before birth due to the increasingly frequent use of fetal echocardiography.

However, we unfortunately still see complex congenital cardiopathies diagnosed late, often when signs and symptoms of severe PH are already manifesting, hindering the total correction of the anomaly. With a lack of reference centers in congenital cardiopathies in Brazil for establishment of early diagnosis and treatment, there are still patients with PH without access to an ideal and definitive surgical treatment.

Progression of PH occurs particularly in patients with large left-right shunts. It is worth noting that the structural changes in the pulmonary circulation are histologically similar to those seen in other forms of primary PH^1^. The presence of large intracardiac communication and ductus arteriosus accelerate the progression of the pulmonary vascular disease^8^.

These patients present clinically with cyanosis, and most are NYHA functional class IV and unable to undergo definitive correction due to elevated levels of pulmonary pressure and vascular resistance. Some centers are trying and testing pharmacological treatment with sildenafil in these patients. However, the high cost of this treatment, lack of standardization by the Unified Health System (*Sistema Único de Saúde, *SUS) and use by only a few institutions provide no scientific evidence to justify its widespread use in this type of patient. (Regarding pharmacological treatment, sildenafil was only approved by the FDA for the treatment of PH patients in 2005, and even then, only for adult patients. Only 1 patient underwent surgery after this date, in 2011. This patient was on sildenafil on his last follow-up in 2014. There was no preoperative pharmacological preparation or postoperative pharmacological treatment intended for PH patients operated on with this technique in the 1990s, the period in which 17 of the 21 surgeries were performed. Only tests with nitric oxide and 100% oxygen during diagnostic catheterization and inhaled nitric oxide after surgery were available. The technique was proposed due to lack of other forms of preoperative care and postoperative treatment.)

Considering that there is no current evidence of treatment for patients in the pediatric age group with severe PH and the fact that the guidelines are empirically based on experts recommendations^1^, the palliative Senning procedure should be considered in patients with late diagnosis, when severe pulmonary vascular disease is already established.

Historically, operations at the atrial level were the first truly effective surgical procedures in the treatment of TGA^9^. The technique proposed by Mustard was the procedure of choice for correction of simple TGA from 1965 to 1982^3^. However, a 1982 survey conducted in several institutions showed a high incidence of complications caused by the synthetic flaps used in the procedure^10,11^. Since the Senning procedure is only performed with autogenous tissues, it has allowed most patients to reach adulthood, with a survival rate of 88% after 20 years and with a late mortality of 9.4% according to Roubertie et al^12^.

It should be noted that arterial correction is still the treatment of choice in the neonatal period in patients with a diagnosis of TGA with or without VSD. These patients are operated on with the technique successfully performed for the first time by Jatene in 1975^13^. After the decade of 1980, this became the surgery of choice by most centers specialized in congenital cardiopathies^11^.

Patients with an intracardiac shunt with increased PVR are unable to be promptly referred to surgical correction of the anomaly, and in many centers are treated with pulmonary vasodilators prior to the surgery. It was previously believed that the early correction of the heart defect would result in regression of the pulmonary vascular abnormalities^6^, regardless of the degree of arterial remodeling^14^. However, wait for the regression of pulmonary lesions only with surgical repair is not recommended, wherein the combination of pharmacological treatment (sildenafil and/or bosentan) has been used in the management of these patients, even in the absence of widespread evidence-based recommendation for this type of approach^1,15^.

Cardiac catheterization is essential to define treatment in patients with PH. Tests with inhaled 100% oxygen and/or nitric oxide are important in defining management. The definition of severe PH is often arbitrary. A PVR of 10 to 12 U.m^2^ or greater is generally considered severe. The presence of advanced grades in the Heath and Edwards histological classification is often considered irreversible^6^. In patients aged 1 to 2 years presenting reduced PVR with inhaled 100% oxygen, the Senning procedure with VSD closure may be considered. However, in patients with an inadequate response to inhaled 100% oxygen, the procedure of choice would be the palliative Senning procedure^5^.

Hemodynamic studies have shown that almost all patients above the age of 1 year with a diagnosis of TGA and large VSD have a significant increase in PVR. Fourteen of the 21 patients operated on at InCor-HCFMUSP were older than 1 year. In contrast, the increase in PVR was a rare finding in older children with TGA and intact ventricular septum^8^.

Newfeld et al^8^ have shown that patients with pulmonary pressure of 50 mmHg or greater and pulmonary biopsies grade 4 or greater were older than 1 year. In contrast, all patients with pulmonary pressure of 50 mmHg or greater with a pulmonary biopsy grade below 4 were younger than 1 year of age^8^. In this study, all patients with a grade 4 biopsy were older than 1 year, and of the 6 patients younger than 1 year at the time of the surgery, 4 presented a grade 2, 1 a grade 1, and 1 a grade 3 biopsy. For the decision of treatment type, age is an important factor in view of the complications associated with longer exposure of the pulmonary parenchyma in cardiopathies with hyperflow.

The development of severe pulmonary vascular disease remains one of the major concerns in patients with TGA, and its occurrence is often considered a contraindication for surgical correction. Histological studies have shown that a rapid progression of the pulmonary vascular disease may occur in TGA patients, particularly in those with non-restrictive VSD^8^. Ferencz^16^ reported early and severe hypertensive changes in biopsies of pulmonary arteries in 106 TGA patients. He noted that the lung lesions increased in severity with the increase in VSD size^16^. Many patients older than 1 year with a diagnosis of TGA with VSD and mean pulmonary pressure of 50 mmHg or greater have established pulmonary vascular disease grade 4 on Heath and Edwards' classification^8^. Of the patients operated on at InCor, 66% had a non-restrictive VSD, and 6 had patent ductus arteriosus causing hemodynamic repercussion.

According to the classic work of Heath et al^17^, when specific histological changes emerge on the pulmonary vasculature of patients with TGA with large communications, PH will not regress until the defect is corrected. These authors also reported that a pulmonary biopsy grade 4 or greater is usually indicative of irreversible pulmonary vascular disease and that the pulmonary pressure levels were unlikely to decrease unless surgical treatment was performed. Pulmonary vascular disease of advanced grade (above 3) also increased significantly the risk associated with surgery and death due to low output, which occurred in the immediate postoperative period^17^. Even after surgical correction, PH may still progress along with the disease. Microscopic studies of these patients' lungs showed biopsies grade 4 or greater in the majority of the cases^18^.

In an attempt to contain the advance of the PH or improve the arterial oxygen saturation, palliative procedures are a therapeutic option. In 1950, Blalock and Hanlon published a procedure that allowed mixing of the pulmonary and systemic circulations with the establishment of an interatrial communication. This was the first palliative procedure to allow survival of patients with TGA and restrictive intracardiac communications. The Rashkind technique has now replaced the previous procedure with enlargement of the foramen ovale with a balloon catheter^11^. Pulmonary artery banding has also been advocated to protect the lungs against the development of pulmonary vascular disease in patients with TGA and VSD, particularly in those younger than 6 months. A persistent large ductus arteriosus should also be treated. In patients with large septal defects, treatment of the ductus arteriosus, pulmonary artery banding, or corrective surgery with closure of the VSD should be performed up to the age of 6 months to prevent progressive pulmonary vascular disease^8^.

There are currently four indications for the Senning procedure. The first is in children with isolated TGA presenting after the neonatal period, in which the left ventricle would already be misadjusted and unable to support the systemic circulation with the Jatene procedure. The second is as a palliative method in patients with a pulmonary vascular disease associated with VSD. The third indication is for patients with corrected TGA. In this case, both the venous and arterial switch are required to create a concordant ventricle (double switch). The fourth is in the presence of rare isolated ventricular inversion. In this situation, there is an atrioventricular discordance with a ventriculoarterial concordance^11^.

Burkhart et al^5^ have shown in a study with 28 patients, operated on at the Mayo Clinic in Rochester and at the Hospital for Sick Children in Toronto, that there was a 23% increase in oxygen saturation, a significant decrease in hematocrit, and improvement in NYHA functional class III and IV to I and II after atrial palliative surgery in patients with severe PH. The Mustard procedure was performed in 25 patients and the Senning procedure in 3^5^. These improvements were also found in all patients operated on with the Senning procedure in our study, with a mean 12.9 points decrease in hematocrit, 30.4 points increase in pulse oximetry, and improvement in functional class. Humes et al^19^ also found a significant decrease in hemoglobin levels and increase in mean oxygen saturation from 64% to 85% after 9 years of follow-up^19^.

In a series of 132 Senning cases with 20 years of follow-up, Roubertie et al^12^ showed a 5.3% mortality within the first 30 thirty days and a 9.6% late mortality. Senning reported that 6% of the patients died due to systemic ventricular failure after 10 years of progression, and Cochrane et al^20^ reported this occurrence in 10% of the patients after 7 years. Right ventricular dysfunction is a well-known late complication of the Senning procedure and is described in almost all studies. The rate of right ventricular dysfunction may be as high as 48% in simple TGAs and 61% in complex TGAs at 15 years of follow-up^12^. It is worth noting that in previous studies the patients had normal pulmonary pressures. Mortality due to ventricular failure occurred within 30 days of follow-up in our series, accounting for 6 of the 10 deaths. Of the 21 patients who were operated on, 11 were discharged from the hospital, and 1 died at home due to an unknown cause after 18 months. Greater rates of sudden death have been reported in patients undergoing the Mustard procedure when compared with those undergoing the Senning procedure^9^.

Rhythm disturbances are the most common causes of morbidity in the first few days after surgery. Junctional rhythm is present in 56% and TAVB in 6% of the operated patients. The late follow-up showed that 65% progressed with brief episodes of junctional rhythm and 38% with sinus bradycardia^3^. Rhythm abnormalities occurred in only 2 patients in our series. One patient presented transient TAVB that soon improved and returned to sinus rhythm, and another patient presented junctional rhythm. Arrhythmias may be explained by reentrant mechanisms caused by suture lines in the atrium, whereas sinus node dysfunction may occur due to direct injury of the node or its artery^8^.

Some late complications may be observed, many related to technical aspects of the surgical correction such as obstruction of the superior vena cava in 10% of the cases, obstruction of the inferior vena cava in 2%, interatrial leaks, obstruction of the pulmonary veins, atrial arrhythmias (sinus node dysfunction), right ventricular dysfunction, and tricuspid insufficiency^21^, this last probably due to annular dilatation as a consequence of right ventricular dysfunction^9^. Baffle stenosis or leak was the main complication in 5% of the patients operated on in Toronto, and also the most frequent reason for reoperation^22^. During follow-up in our study, we found 1 case of cava baffle stenosis and 5 cases of tricuspid valve dysfunction. Reoperations are related to systemic venous or pulmonary venous obstruction^3^. Sarkar et al^23^ found a lower incidence of reoperation for intra-atrial baffle abnormalities in patients operated with the Senning procedure. They occurred in 12% of the 226 survivors undergoing the Mustard procedure and in 2% of the 132 survivors undergoing the Senning procedure^23^.

The palliative Senning procedure aims at improving the quality of life in critical patients unable to undergo another surgical treatment or improve with pharmacological therapy, since high levels of pulmonary pressure increase the risk of premature mortality and worsen the quality of life of the few survivors. The group of patients included in this study had elevated early mortality with low output as the main cause. The hypoxemia in these patients, who survive in a regimen of overload both in the systemic right ventricle as well as in the pulmonary left ventricle, aggravate the function of the ventricles. This has also been reported by Burkhart et al^5^ who found low output as the main cause of early mortality in 5 of the 6 deaths within the first 30 days^5^. Low output was also the leading cause in our series, accounting for 6 of the 10 initial deaths. However, the survival rate found in our study after 19 years of follow-up was superior: 52.3% versus 46.4%. The fact that 7 of the 10 deaths occurred more than 20 years ago may be associated with the few therapeutic resources existing at that time. The use of nitric oxide as postoperative treatment was not feasible in all patients in the initial series due to the absence of this resource in our institution in the early 1990s. Lack of improved postoperative support, which contrasts to the support currently available, may have influenced the early mortality in the first operated patients.

## Conclusion

The palliative Senning procedure improved arterial oxygen saturation, reduced polycythemia, and provided a better quality of life to patients with TGA and VSD or Taussig-Bing DORV who had severe PH, were considered inoperable, and had a poor prognosis. Our study also showed that pulmonary lesions of more advanced grades are predominant in patients who were operated on after the age of 12 months. This confirms the need for surgical treatment as early as possible.
